# Supplementary figures and images for: Automatic feature selection for performing Unit 2 of vault in wheel gymnastics
Source: PLoS One. 2023 Jun 23;18(6):e0287095. doi: 10.1371/journal.pone.0287095 (PMC10289312; doi:10.1371/journal.pone.0287095)

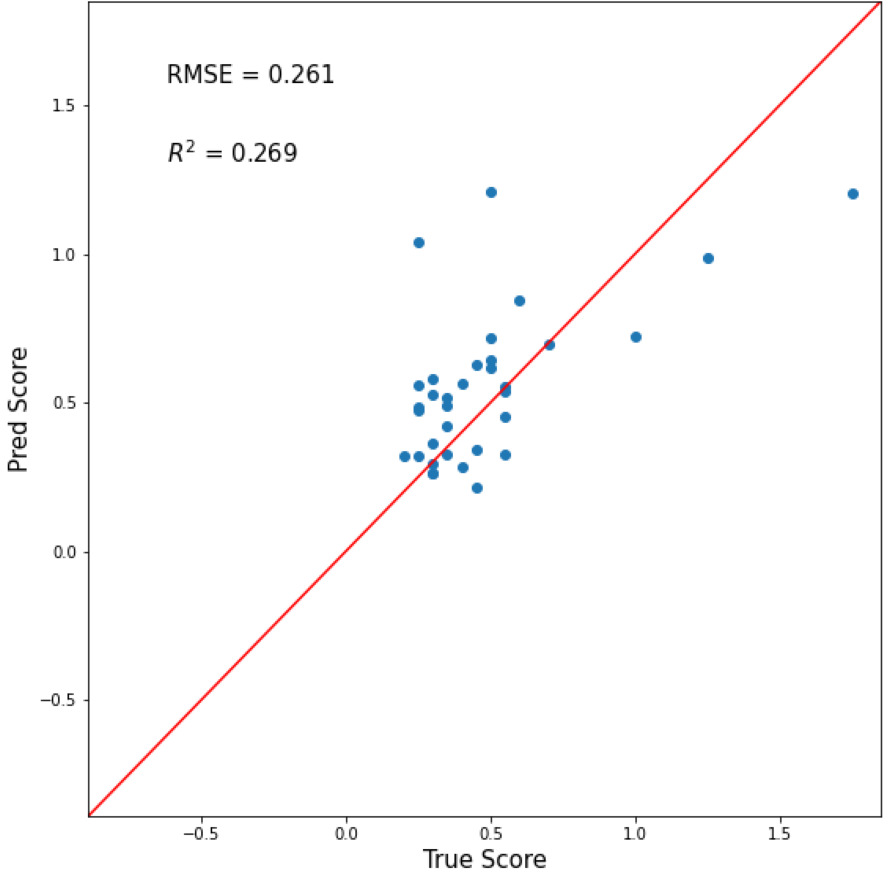

Supplement: S1 Fig — This model consisted of an input layer with 21 units, an LSTM layer with 64 internal ones, and a dense layer of 1 output with the identity function (for more details of the hyperparameters, see S1 Table). The inputs of training and test data were identical to those in the main body of our manuscript, with standardization. (TIF) [file pone.0287095.s001.tif]

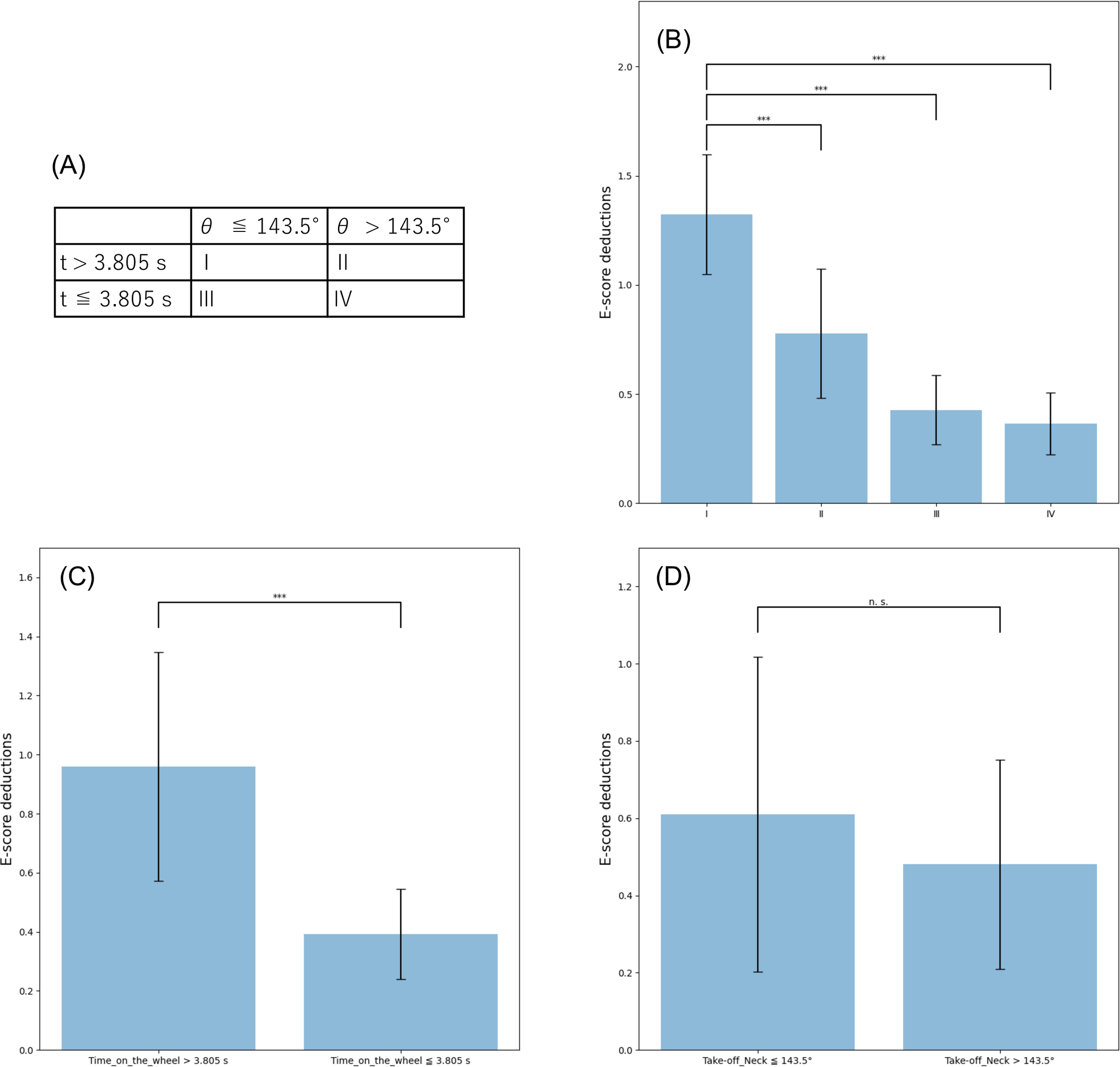

Supplement: S2 Fig — (A) Four groups of the vault performances divided by whether they met the two conditions derived by Random Forests: time on the wheel over 3.805 s, and neck angle in the take-off position within 143.5°. (B) Multiple comparisons of the E-score deductions for the vault performances when (I) t > 3.805 s and θneck ≤ 143.5° with those of the other conditions (II)—(IV) (Dunnett’s test, p < 0.001). (C) Comparison of the E-score deductions between two groups divided only by whether t > 3.805 s or not (Mann–Whitney U test, p < 0.001). (D) Two-group comparison when θneck ≤ 143.5° and not (Mann-Whitney U test, p ≥ 0.05). (TIF) [file pone.0287095.s002.tif]

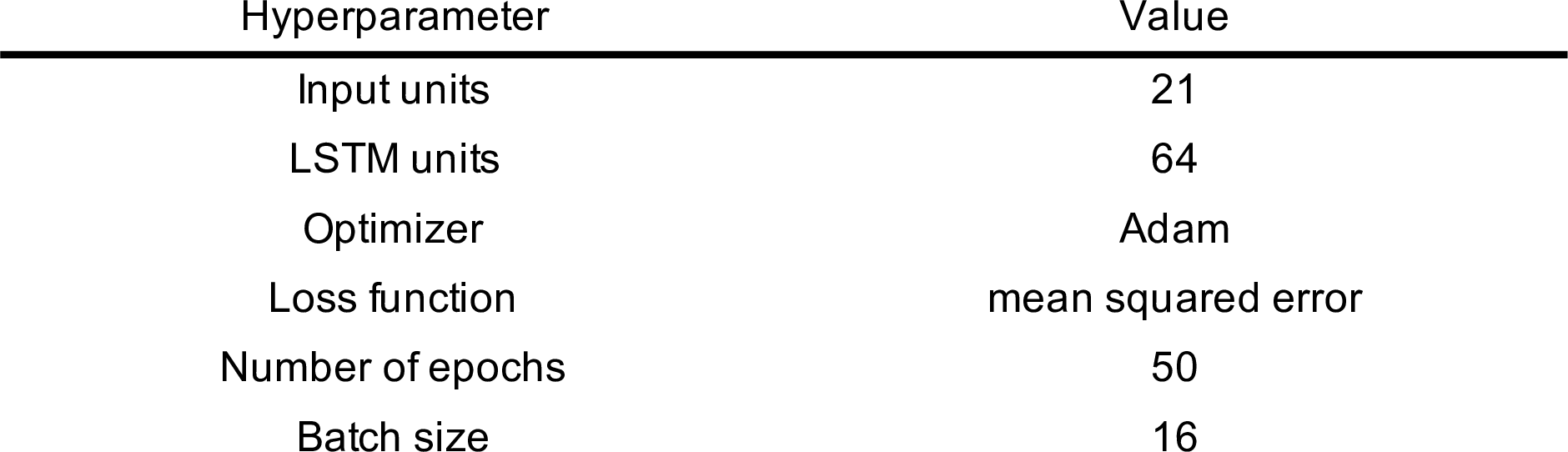

Supplement: S1 Table — (TIF) [file pone.0287095.s003.tif]
