# Supplementary material for: Automatic feature selection for performing Unit 2 of vault in wheel gymnastics
Source: PLoS One. 2023 Jun 23;18(6):e0287095. doi: 10.1371/journal.pone.0287095 (PMC10289312; doi:10.1371/journal.pone.0287095)
Supplement: Attachment — Submitted filename: Response_to_Reviewers_Kitajima_PLOSONE2023.pdf [file pone.0287095.s004.pdf]

Academic Editor comments

Thank you for submitting your manuscript to PLOS ONE. After careful consideration, we feel that it has merit but does not fully meet PLOS ONE's publication criteria as it currently stands. Therefore, we invite you to submit a revised version of the manuscript that addresses the points raised during the review process.

[Our response] Thank you for the positive comments about our manuscript. We will do our best to get it published.

Journal Requirements

1. Please ensure that your manuscript meets PLOS ONE's style requirements, including those for file naming. The PLOS ONE style templates can be found at https://journals.plos.org/plosone/s/file?id=wjVg/PLOSOne_formatting_sample_main_body.pdf and https://journals.plos.org/plosone/s/file?id=ba62/PLOSOne_formatting_sample_title_authors_affiliations.pdf

[Our response] We have checked that our manuscript meets the PLOS ONE's style requirements. We have also listed Supporting Information captions at the end of the manuscript in a section titled "Supporting information" (p. 13, line 484 – line 491).

2. Thank you for stating the following financial disclosure:
"NO: The funders had no role in study design, data collection and analysis, decision to publish, or preparation of the manuscript."

At this time, please address the following queries:

a) Please clarify the sources of funding (financial or material support) for your study. List the grants or organizations that supported your study, including funding received from your institution.

b) State what role the funders took in the study. If the funders had no role in your study, please state: “The funders had no role in study design, data collection and analysis, decision to publish, or preparation of the manuscript.”
c) If any authors received a salary from any of your funders, please state which authors and which funders.
d) If you did not receive any funding for this study, please state: “The authors received no specific funding for this work.”

Please include your amended statements within your cover letter; we will change the online submission form on your behalf.

[Our response] The authors received no specific funding for this work. We have included our amended statements within our revised cover letter as “The authors received no specific funding for this work.”

3. In your Data Availability statement, you have not specified where the minimal data set underlying the results described in your manuscript can be found. PLOS defines a study's minimal data set as the underlying data used to reach the conclusions drawn in the manuscript and any additional data required to replicate the reported study findings in their entirety.
All PLOS journals require that the minimal data set be made fully available. For more information about our data policy, please see http://journals.plos.org/plosone/s/data-availability.

Upon re-submitting your revised manuscript, please upload your study’s minimal underlying data set as either Supporting Information files or to a stable, public repository and include the relevant URLs, DOIs, or accession numbers within your revised cover letter. For a list of acceptable repositories, please see http://journals.plos.org/plosone/s/data-availability#loc-recommended-repositories. Any potentially identifying patient information must be fully anonymized.

Important: If there are ethical or legal restrictions to sharing your data publicly, please explain these restrictions in detail. Please see our guidelines for more information on what we consider unacceptable restrictions to publicly sharing data: http://journals.plos.org/plosone/s/data-availability#loc-unacceptable-data-access-restrictions. Note that it is not acceptable for the authors to be the sole named individuals responsible for ensuring data access.

We will update your Data Availability statement to reflect the information you provide in your cover letter.

[Our response] We have included the Data Availability statement within our revised cover letter as “All data are available at the following link. https://drive.google.com/drive/folders/1eQpiWy6cjM07_QN6w5bXC8CLez02pzY1?usp=sharing”.

4. We note that Figure 3 in your submission contain copyrighted images. All PLOS content is published under the Creative Commons Attribution License (CC BY 4.0), which means that the manuscript, images, and Supporting Information files will be freely available online, and any third party is permitted to access, download, copy, distribute, and use these materials in any way, even commercially, with proper attribution. For more information, see our copyright guidelines: http://journals.plos.org/plosone/s/licenses-and-copyright.

We require you to either (1) present written permission from the copyright holder to publish these figures specifically under the CC BY 4.0 license, or (2) remove the figures from your submission:

a. You may seek permission from the original copyright holder of Figure 3 to publish the content specifically under the CC BY 4.0 license.

We recommend that you contact the original copyright holder with the Content Permission Form (http://journals.plos.org/plosone/s/file?id=7c09/content-permission-form.pdf) and the following text:
“I request permission for the open-access journal PLOS ONE to publish XXX under the Creative Commons Attribution License (CCAL) CC BY 4.0 (http://creativecommons.org/licenses/by/4.0/). Please be aware that this license allows unrestricted use and distribution, even commercially, by third parties. Please reply and provide explicit written permission to publish XXX under a CC BY license and complete the attached form.”

Please upload the completed Content Permission Form or other proof of granted permissions

as an ""Other"" file with your submission.

In the figure caption of the copyrighted figure, please include the following text: “Reprinted from [ref] under a CC BY license, with permission from [name of publisher], original copyright [original copyright year].”

b. If you are unable to obtain permission from the original copyright holder to publish these figures under the CC BY 4.0 license or if the copyright holder’s requirements are incompatible with the CC BY 4.0 license, please either i) remove the figure or ii) supply a replacement figure that complies with the CC BY 4.0 license. Please check copyright information on all replacement figures and update the figure caption with source information. If applicable, please specify in the figure caption text when a figure is similar but not identical to the original image and is therefore for illustrative purposes only.

[Our response] We have obtained permission from the original copyright holder of Figure 3 to release the content under a CC BY 4.0 license in accordance with the above requirement (1), a, and uploaded the completed Content Permission Form as an "Other" file. We have also included the following text in our revised manuscript: “Reprinted from [22] under a CC BY license, with permission from Sorao Kosaka, original copyright 2020” in the figure 3 caption (p. 5, after line 153).

5. We note that Figures 1, 2 and 7 includes an image of a participant in the study.
As per the PLOS ONE policy (http://journals.plos.org/plosone/s/submission-guidelines#loc-human-subjects-research) on papers that include identifying, or potentially identifying, information, the individual(s) or parent(s)/guardian(s) must be informed of the terms of the PLOS open-access (CC-BY) license and provide specific permission for publication of these details under the terms of this license. Please download the Consent Form for Publication in a PLOS Journal (http://journals.plos.org/plosone/s/file?id=8ce6/plos-consent-form-english.pdf). The signed consent form should not be submitted with the manuscript, but should be securely filed in the individual's case notes. Please amend the methods section and ethics statement of the manuscript to explicitly state that the patient/participant has provided consent for publication: “The individual in this manuscript has given written informed consent (as outlined in PLOS consent form) to publish these case details”.

If you are unable to obtain consent from the subject of the photograph, you will need to remove the figure and any other textual identifying information or case descriptions for this individual.

[Our response] The specific permission to publish under the PLOS open-access (CC-BY) license has been obtained from the participants in this study. We have included the following text in the revised manuscript: The individuals in this manuscript have given written informed consent (as outlined in PLOS consent form) to publish these case details (p. 4, lines 128-130).

Additional Editor Comments:

Associate Editor:
Reviewers have found potential in the study. However, there are several concerns that need to be addressed and I concur with the reviewers. I encourage the authors to revise upon reviewers comments and resubmit the manuscript.

[Our response] Thank you for your suggestion. We took the reviewers' comments seriously and incorporated them into the manuscript to the best of our ability.

5. Review Comments to the Author

Please use the space provided to explain your answers to the questions above. You may also include additional comments for the author, including concerns about dual publication, research ethics, or publication ethics. (Please upload your review as an attachment if it exceeds 20,000 characters)

Reviewer #1: In this study, authors proposed a framework to analyze the relationship between the movement features of a wheel gymnast during Unit 2 of the vault event and the E-score deductions from ML.

o Authors mentioned deep network architectures in the abstract. Better to mention the name of the deep learning architecture.

[Our response] Following your suggestion, we added RAFT, the abbreviated name of deep learning architecture, to line 9 of the abstract (p. 1).
We also italicized the names of deep or machine learning algorithms used in this study to make them stand out.

o Authors used deeplabcut to acquire the coordinates of the gymnast's body joints. Nowadays openpose is quite popular for the extraction of body joints. Openpose also estimates based on optical flow.
Noori, Farzan Majeed, et al. "A robust human activity recognition approach using openpose, motion features, and deep recurrent neural network." Scandinavian conference on image analysis. Springer, Cham, 2019.
Cao, Z., et al. "OpenPose: Realtime Multi-Person 2D Pose Estimation Using Part Affinity Fields. In IEEE Transactions on Pattern Analysis and Machine Intelligence." (2019).

[Our response] To ensure the generalization of pose estimation we changed the pose estimation model from DeepLabCut to OpenPose. Previously, we avoided using OpenPose because we mistakenly believed that it could not be used in the sports field due to the following license. "The non-exclusive commercial license cannot be used in the field of Sports.", according to this page. However, we found the following text on the same page: "Just looking for an academic license?" which confirms that there is a license for academic use only. We have reflected this comment in the revised manuscript (p. 6, lines 209 - 218) and in the figure 5 caption (p. 7, after line 226).

o If some joints of the frames were missing, how authors incorporated with such sort of issue, which is common in body joints detection through computer vison.

[Our response] There was no missing detection of body joints in this study, and if there had been, we would have discarded the data. The possibility of missing detection of body joints is small because we carefully selected the recording conditions and placed the cameras at distances that would allow us to capture the gymnasts. We have reflected this comment in the revised manuscript (p. 6, line 220 and p.7, lines 221 - 223).

o It is time series data, why authors preferred random forests why not state-of-the LSTMs, in that

case it would be end-to-end deep learning approach.

[Our response] Before the first submission of our manuscript, we had also considered an end-to-end deep-learning approach in which the inputs of the LSTM network to estimate the E-score deductions were the time-series data of each body joint coordinate obtained with a pose estimation model. This approach, however, would require at least thousands of performance videos since the time on the wheel could fluctuate at each performance, and it was impractical to acquire such an enormous dataset.

To select the significant features of motions related to the E-score deductions from a limited number of data, we first focused on the critical phases mentioned in the general vault regulations by implementing the automatic extraction of the video frames required for the scoring.
We then narrowed down the candidates by reference to the value of feature importance output from the decision-tree-based ensemble model, which did not require thousands of samples or preprocessing like normalization and standardization that deep learning did.

For comparison, we included in the Supporting Information of our manuscript the results of estimating the E-score deductions using an LSTM network input with the same 21 features as those of the random forest and trained with the same training data.
As shown in S1 Figure, the LSTM only achieved lower accuracies than the Random Forests in terms of either R^2 or RMSE.

We have reflected the above comments in the revised manuscript (p. 8, lines 276 - 278) and in the S1 Fig caption (p. 13, lines 486 - 490).

o Authors mentioned *we extract the four frames required for scoring the execution using deep learning-based computer-vision techniques* . Please specify the model/technique here or mention the section number about further details, it was a bit challenging to follow.

[Our response] We have reflected this comment in the Fig 2 caption: “we extract the four frames required for scoring the execution using deep learning-based models; RAFT [13], and XMem [14]. Second, from the extracted video frames, we obtain the joint coordinates of the on-screen gymnast using a pose-estimation model (BODY 25) of OpenPose [15] then calculate the joint angles in each frame. Finally, on the basis of the joint angles, we estimate the E-score deductions during Unit 2 using Random Forests [16], which enables us to automatize the feature selection by reference to the nonzero values of feature importances. More details of these three processes are in the fourth to sixth subsections of Materials and Methods (see also Figs. 4－6)” (p. 3, after line 84).

o Title of the manuscript shows authors mention selection of features. I am failing to understand that the selection was manual or automatic.

[Our response] Our proposed framework automatically works to feature selection once the videos of a wheel gymnast performing the tuck-front somersault performance are uploaded. As additionally noted in the caption of Fig. 6 (B), many features have zero feature importance values. In other words, the Random Forests automatically select a small number of motion features with nonzero feature importance values related to the E-score deductions. When further narrowing down the candidates into one or two, as in the discussion, we need to manually select features with prominent feature importance values. We have reflected this comment in the Fig 2 caption: "Finally, on the basis of the joint angles, we estimate the E-score deductions during Unit 2 using Random Forests [16], which enables us to automatize the feature selection by reference to the nonzero values of feature importances" (p. 3, after line 84) and in the Random Forests section: "For automatic feature selection to avoid significant E-score deductions, we measured each feature importance with the threshold of 0.1 of the trained model" (p. 8, lines 260 - 262). We have also changed the title to "Automatic feature selection for performing Unit 2 of vault in wheel gymnastics".

o Abstract line 5: it should be its not his. Please proofread the whole manuscript.

[Our response] We have reflected this comment by changing the word "his" to "its" (p. 1, line 6). We have also corrected terminology throughout the manuscript.

Reviewer #2: The authors of the manuscript present a framework for analyzing the relationship between the movement features of a wheel gymnast during the mounting phase of Unit 2 of the vault event and execution (E-score) deductions, using a machine learning approach. They utilized gymnastics rules and machine learning techniques to determine the E-score deductions. According to the authors, this is the first study of its kind to quantify the E-score deductions in wheel gymnastics using a computer vision approach.

Some areas for improvement in the manuscript include increasing the number of subjects, as the

results of the proposed framework would be stronger if they were based on the analysis of multiple gymnasts, allowing for statistical analysis and the application of null hypothesis to assess the significance of the method. The quality of the figures, specifically Figure 6, could also be improved by enlarging the font size of the x- and y-axes for better readability. Additionally, the manuscript's sentence structure and use of academic-style English could be improved.

[Our response] In response to your suggestion, we quickly recruited another male wheel gymnast, who had a similar physical size and gymnastic skill to the first gymnast. At the same time, we filed a research ethics application to our university and subsequently received approval for additional experiments with human subjects. We added the new approval number of 58 to the subsection of Participants in our manuscript (p. 4, line 131). We then recorded eight videos of the second gymnast performing a tuck-front somersault over three days. We merged them into a test dataset and analyzed them with the proposed framework, founding that they were also predictable with high accuracies of both R^2 and RMSE, as shown in the new Fig. 6 (A).

In the new Fig. 6, we enlarged the font sizes and replaced the tree samples with easier-to-grasp ones.

Since the journal also requested that we revise our manuscript to meet PLOS ONE's style template, we holistically readjusted the sentence structure.

We have reflected this comment in the revised manuscript (p. 8, lines 273 - 275).
