# Supplementary material for: Automatic feature selection for performing Unit 2 of vault in wheel gymnastics
Source: PLoS One. 2023 Jun 23;18(6):e0287095. doi: 10.1371/journal.pone.0287095 (PMC10289312; doi:10.1371/journal.pone.0287095)
Supplement: Attachment — Submitted filename: Response_to_Reviewers_Kitajima_PLOSONE2023.pdf [file pone.0287095.s005.pdf]

Academic Editor comments

Thank you for submitting your manuscript to PLOS ONE. After careful consideration, we feel that it has merit but does not fully meet PLOS ONE's publication criteria as it currently stands. Therefore, we invite you to submit a revised version of the manuscript that addresses the points raised during the review process.

Some minors revisions are still required. R2 has suggested to include statistical test to verify the claims and I concur with him.
Please ensure that your decision is justified on PLOS ONE's publication criteria and not, for example, on novelty or perceived impact.

[Our response] Thank you for the positive comments about our manuscript. We will do our best to get it published.

Journal Requirements

1. Please review your reference list to ensure that it is complete and correct. If you have cited papers that have been retracted, please include the rationale for doing so in the manuscript text, or remove these references and replace them with relevant current references. Any changes to the reference list should be mentioned in the rebuttal letter that accompanies your revised manuscript. If you need to cite a retracted article, indicate the article's retracted status in the References list and also include a citation and full reference for the retraction notice.

[Our response] We have checked that our reference list is complete and correct. For ease of finding also the article written in Japanese (reference number 12), we added the DOI to it (p. 13, lines 462-463). The following references (after number 38) in the "Revised Manuscript with Track Changes" were removed from the reference list in the "Manuscript" because they are no longer used in this study: DeepLabCut (p. 15, lines 545-547), DeeperCut (p. 15, lines 548-550), ResNet (p. 15, lines 551-553), DeepLabCut implementation (p. 15, lines 554-556), and dtreeviz (p. 15, lines 557-559). As for reference number 33 in the manuscript, we aligned the spacing (p. 14, lines 524-525).

We also have added the following references in our manuscript after the first submission: OneFormer (p. 13, lines 483-485), Dataset repository (p. 13, lines 486-489), schematic representation vault (p. 13, lines 490-491), Comparing the Quality of Human Pose Estimation

with BlazePose or OpenPose (p. 14, lines 507-509), Openpose 1.7.0 Demo (p. 14, lines 510-513), Long short-term memory (p. 14, lines 524-525), A Multiple Comparison Procedure for Comparing Several Treatments with a Control (p. 14, lines 529-531), scipy.stats.dunnett (p. 14, lines 532-535), On a Test of Whether one of Two Random Variables is Stochastically Larger than the Other (p. 14, lines 536-538), scipy.stats.mannwhitneyu (p. 15, lines 539-542).

Responses to the review comments

Reviewer #1: The authors have revised the paper, I am happy to accept it for the publication in current form.
I have one minor suggestion, regarding LSMTs, mention the issue in limitations. It would be beneficial for readers.

Congratulations!

[Our response] Thank you for your suggestion. We have added the LSTM issue in limitations of our manuscript (p. 11-12, lines 413-420).

Reviewer #2: I commend the authors for their hard work and commitment in improving the manuscript. They conducted further data acquisition, processing, and analysis using the proposed framework on an additional gymnast. In addition, they have enhanced the quality of the figures and the overall structure of the manuscript.
However, the statistical significance analysis still requires improvement. It is recommended that an appropriate statistical significance test or null-hypothesis test, such as Student's t-test or ANOVA, should be employed to calculate the performance of e-score to support the proposed method and claims.

[Our response] In response to your suggestion, we conducted several statistical significance tests and reflected the results in Discussion (p. 10, lines 337-373), the new Fig. 7 (p. 10, after line 373), and S2 Fig (p. 15, lines 551-559).
We confirmed the statistical analysis supported our claims from the proposed methods based on the machine-learning algorithms. The previous Fig. 7 was accordingly changed to Fig 8 (p. 11, after line 401), and the content is the same.
